# Hybridization in natural sympatric populations of *Dermacentor* ticks in northwestern North America

**DOI:** 10.1002/ece3.496

**Published:** 2013-02-14

**Authors:** A Araya-Anchetta, G A Scoles, J Giles, J D Busch, D M Wagner

**Affiliations:** 1 Center for Microbial Genetics and Genomics, Northern Arizona University1298 S. Knoles Dr., ARD Building, Flagstaff, AZ, 86011; 2 USDA, ARS, Animal Disease Research Unit, Washington State University3003 ADBF, PO Box 646630, Pullman, WA, 99164

**Keywords:** *D. variabilis*, *Dermacentor andersoni*, hybridization, population structure, ticks

## Abstract

Hybridization in ticks has been described in a handful of species and mostly as a result of laboratory experiments. We used 148 AFLP loci to describe putative hybridization events between *D. andersoni* and *D. variabilis* in sympatric populations from northwestern North America. Recently, *D. variabilis* has expanded its range westward into the natural range of *D. andersoni*. Using a sample of 235 *D. andersoni* and 62 *D. variabilis*, we identified 31 individuals as putative hybrids: four F_2_ individuals and 27 backcrosses to *D. andersoni* (as defined by NewHybrids). We found no evidence of hybrids backcrossing into *D. variabilis*. Furthermore, all hybrids presented 16S mtDNA signatures characteristic of *D. andersoni,* which indicates the directionality of the hybrid crosses: female *D. andersoni* × male *D. variabilis*. We also discovered 13 species-specific AFLP fragments for *D. andersoni*. These loci were found to have a decreased occurrence in the putative hybrids and were absent altogether in *D. variabilis* samples. AFLP profiles were also used to determine the levels of genetic population structure and gene flow among nine populations of *D. andersoni* and three of *D. variabilis*. Genetic structure exists in both species (*D. andersoni*, Φ_ST_ = 0.110; *D. variabilis,* Φ_ST_ = 0.304) as well as significant estimates of isolation by distance (*D. andersoni, ρ* = 0.066, *P* = 0.001; *D. variabilis,* ρ = 0.729, *P* = 0.001).

## Introduction

An estimate of 10% of animal species and 25% of plant species are found to be capable of hybridization and/or introgression, with some taxa more prone than others (Mallet 2005). Hybridization has been traditionally viewed as a maladaptive event because it is expected to break apart co-adapted gene complexes important for survival. These are indeed negative outcomes that render certain hybrids less fit and less likely to be observed in nature (Ohta 1980).

However, successful hybridization between species has been described in numerous wild and domesticated plants and animals (for reviews see Arnold 1992, 2004), breaking the traditional tenet. Furthermore, hybridization and gene introgression have been described as major sources of genetic variation among individuals and within populations (Barton 2001; Grant et al. 2005), and as such are forces for evolutionary change (Anderson and Stebbins 1954; Arnold 2004). The new gene combinations that result become potential targets for natural selection (Anderson and Stebbins 1954; Dowling and Secor 1997; Mallet 2005, 2007). In this manner, populations or species may mix successfully and become capable of adapting to new ecological niches, or hybrids may backcross to one or either parent species and broaden the cumulative genetic variation in a parent species.

The effects of hybridization between arthropod vectors of disease remain largely unexplored. Inter-species genetic exchange may impact the biology of the vectors, the interaction with their hosts, and even the pathogens they transmit. For instance, a range expansion of the malaria vector *Anopheles gambiae* into more arid environments of Africa is considered to be a result of gene introgression between *A. gambiae* and *A. arabiensis*, (Besansky et al. 2003). Similarly, hybridization between two biotypes of *Culex pipiens* mosquitoes allowed for a broadening of the host preferences along a hybrid zone (Byrne and Nichols 1999; Kilpatrick et al. 2007). These two examples of hybridization between vectors suggest that admixture has been beneficial for these species and may have significant consequences for transmission of vector-borne pathogens. Range expansion and broader host preferences could lead to more rapid spread of the pathogens they carry. More information is needed to deepen our understanding of other effects hybridization may have in the three-way interactions of pathogen, vector, and host.

Ticks serve as vectors for a wide variety of disease agents, and are second only to mosquitoes in their importance to humans in this role. Laboratory experiments using a handful of *Dermacentor* and *Rhipicephalus* (*Boophilus*) species have shown hybridization to be possible in some of these species (Graham et al. 1972; Oliver et al. 1972; Gladney and Dawkins 1973; Davey et al. 1991). However, hybrid ticks have rarely been found in the wild (Rees et al. 2003). This may be due to the difficulty in identifying hybrids or the fact that F_1_ hybrid ticks and backcrosses are often morphologically undifferentiated from either parent species (Barton 2001; Rees et al. 2003). When morphology is ambiguous, molecular methods can provide a powerful means of detecting cryptic hybridization.

In this study, we use molecular markers to report the occurrence of hybridization between *D. andersoni* and *D. variabilis*, which has only been previously reported in laboratory experiments (Oliver et al. 1972). These tick species are relevant because they are vectors of the pathogens that cause disease in humans (Rocky Mountain spotted fever, tularemia, Colorado tick fever, and others) and animals (Anaplasmosis). We suspected hybridization between these two species, given that *D. variabilis* is currently undergoing a westward expansion (Stout et al. 1971) into the natural range of *D. andersoni* driven by the movement of humans and their pets (particularly dogs). We use genetic markers to explore whether equal genetic mixing occurs among parent species, or if one-way introgression into one of the parent species has occurred. Furthermore, we also characterize the population genetic structure of *D. andersoni* along its natural range in the northwest intermountain region of North America, and report genetic structure in disjunct western populations of *D. variabilis*.

## Materials and methods

### Tick species

*Dermacentor andersoni* (Stiles), the Rocky Mountain wood tick, and *Dermacentor variabilis* Say, the American dog tick, are hard ticks of the family Ixodidae. Both are three-host ticks; each life stage feeds on a different host and molting between stages occurs off of the hosts. Mating occurs on the host and engorged females drop to lay eggs (Sonenshine 1993). Thus, maximum gene dispersal per generation in *D. andersoni* and *D. variabilis* should be determined largely by movement of their mammal hosts. Each tick uses a large variety of host species, with some overlap. Adult *D. andersoni* are found primarily on large herbivores like deer, elk, cattle, horses, and sheep, but they also utilize a variety of other mammals such as bears, dogs, larger rodents (porcupines, marmots, squirrels), and lagomorphs (rabbits, hares, and pika). In contrast, adult *D. variabilis* are found primarily on wild and domestic canids (dogs, coyotes and foxes), as well as on felids, mustelids (badgers and weasels), bears, raccoons, skunks, rabbits, voles, and opossums. They rarely use larger animals like deer, horses, and cattle. The immature stages (larvae and nymph) of these two ticks show almost no overlap in host preferences, with *D. andersoni* using a wider host range that includes many species of chipmunks and ground squirrels and, less frequently, marmots, lagomorphs, voles, *Peromyscus*, and wood rats. Meanwhile, immature *D. variabilis* are found overwhelmingly on voles and rarely on *Peromyscus* mice (Gregson 1956; Strickland et al. 1976; Furman and Loomis 1984; James et al. 2006).

*D. andersoni* is found throughout the Rocky Mountain region of the western US and southern Canada, especially in semiarid sagebrush steppe grasslands (Burgdorfer 1969; James et al. 2006). *D. variabilis* occurs primarily in eastern North America and the Great Plains region, but its range also includes California and a few scattered populations in the western US. It prefers grassy meadows and deciduous forests (Sonenshine 1993). It is commonly found along trails and roads (Burgdorfer 1969). Even though these species differ in their habitat preferences, small sympatric populations of *D. andersoni* and *D. variabilis* are found in the US in Nebraska, North and South Dakota, Montana, and the province of Saskatchewan in Canada (Dergousoff and Chilton 2007). The life cycles of these ticks vary in length. *D. variabilis* can advance through all stages in the term of 1 year in warmer climates, but usually takes 2 years in colder northern locations (Sonenshine 1993). *D. andersoni* ticks live longer (2–3 years) with all life stages capable of overwintering (Burgdorfer 1969; James et al. 2006). In both cases, adults from different generations may overlap in a given population.

### Sample collection

**Figure 1:**
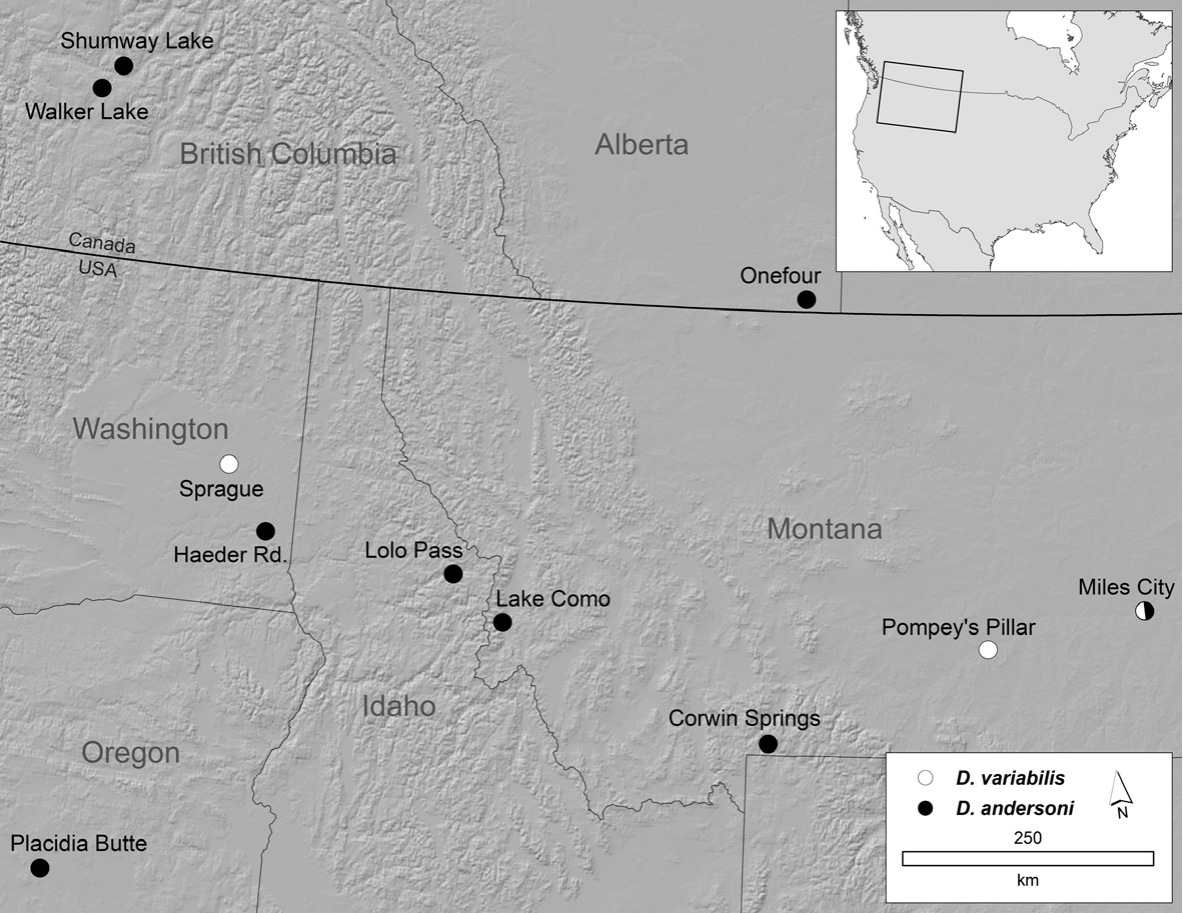
Collection locations of *Dermacentor andersoni* and *D. variabilis* ticks. Sample sizes are provided in Table 2.

Sample collection for *D. andersoni* took place in the states of Washington, Idaho, Oregon, and Montana in the US, and Alberta and British Columbia in Canada, during April–May 2002 and April 2003. *D. variabilis* collections were made during April 2003 and April–June 2004 at sites in Montana and Washington. Samples of each species were collected by dragging a one-meter square piece of white cloth through the vegetation along trails and in areas ticks were expected to be questing. This method and the timing of collection preferentially samples adults. Intact ticks were preserved in 70% ethanol until use for DNA isolation (Scoles 2004). A total of 235 *D. andersoni* adults were collected at nine locations (Fig. 1). One nymph and 63 adult *D. variabilis* specimens were found in three locations (Fig. 1). *D. variabilis* is currently undergoing a westward range expansion in the US (Stout et al. 1971). This expansion has been largely mediated by the movement of pets (mostly dogs) traveling with humans. Although very similar in appearance, *D. andersoni* and *D. variabilis* can usually be separated unambiguously using morphological characters (Yunker et al. 1986). All ticks that were later putatively identified as hybrids were identified as *D. andersoni* at the time of collection based on morphology.

### AFLP analyses

Whole ticks were used for total genomic DNA extractions using the DNeasy Tissue Kit (Qiagen, Germantown, MD) according to the manufacturer's protocol with the modifications described by Scoles (2004). Genetic markers were generated using the amplified fragment length polymorphisms (AFLP) technique described in Vos et al. (1995) and modified by Travis et al. (1996) and Busch et al. (2000). Additional changes to the published protocols included the following: (1) a final concentration of 250 ng/μL of BSA was added to the restriction–ligation (RL) reactions; and (2) each RL and preselective amplification reaction was diluted 1/10 in molecular grade water. To avoid contamination errors, negative controls (molecular grade water instead of DNA template) were included at every step. Possible primer combinations were tested using samples from all populations of both species. Four +3/+4 primer combinations of EcoRI/MseI were used in selective amplifications: ACG/CCAA, AGC/CCAA, ACG/CGAA, and AGC/CGAA (Table 1). EcoR1 primers were fluorescently labeled with HEX® dye (Applied Biosystems, Foster City, CA) for automated detection of fragments. Reaction parameters and PCR conditions used were those in Vos et al. (1995) and modified in Travis et al. (1996).

**Table 1:** AFLP primer combinations including the number of scored fragments for *Dermacentor variabilis* and *D. andersoni*

Primer combination	EcoR1 (3′-NNN)	Mse1 (3′-NNNN)	No. of scored fragments
1	ACG	CCAA	33
2	AGC	CCAA	26
3	ACG	CGAA	47
4	AGC	CGAA	42
Total			148

AFLP fragments were scored against the MapMarker® X-Rhodamine Labeled 50–1000 bp size standard electrophoresed on an ABI 3730 Genetic Analyzer and analyzed with GeneMapper® Software v.4.0 (Applied Biosystems, Foster City, California). To reduce the probability of errors due to homoplasy between loci and/or automated scoring mistakes, a conservative approach to scoring was defined. First, loci were selected when at least one individual possessed a band of ≥1500 relative fluorescent units (RFUs). This step reduced the number of usable fragments by about 75%. Furthermore, it assured that only loci presenting a strong signal were considered for analysis. Second, only loci separated by at least ± 1 bp were scored. This was done to avoid a software bias that consistently scores the taller of two bands within 1 bp of each other. Third, given that PCR favors amplification of small fragments only loci between 100 and 500 bp were scored. This lowered the probability of homoplasy between loci, which is a problem with small size markers (<100 bp) (Caballero et al. 2008). Fourth, once the loci for analysis were selected using the three previous steps, the intensity for band detection across all individuals was relaxed to 100 RFUs to include bands with signals lower than 1500 RFUs. Finally, scores were double-checked visually for errors. Samples with abnormal profiles were discarded and reactions repeated. Only fragments that were polymorphic at the 95% level for all individuals from both species were considered for scoring. The risk of scoring AFLP peaks from host DNA was minimal because we collected questing ticks that would not have fed since before their previous molt and, therefore, would have little or no host DNA present in their guts.

To determine the genotyping error rate, a random set of individuals was duplicated at the second and third steps: (1) a subset of duplicates was started at the preselective amplification stage (step 2) and taken through the rest of the procedure; and (2) a second subset was duplicated only for selective amplifications (step 3). Scores were compared for duplicate samples and the error rate was calculated as the number of differences divided by the number of comparisons (Bonin et al. 2004).

All pairs of loci were tested for linkage disequilibrium (LD) in Arlequin 3.5.1.2 (Excoffier and Lischer 2010). We used sample sizes ≥30 for *D. andersoni* and the two largest samples available for *D. variabilis* (*n* = 19 and 40). If two loci showed LD in at least 50% of the populations tested, then they were considered in LD for the entire dataset.

To confirm the ability of the chosen loci to separate between species, an analysis of similarity (ANOSIM) was performed using Primer v.5.2.9 (Clarke and Gorley 2001). ANOSIM was performed using Nei's genetic distances as calculated in Genalex v.6.3 (Peakall and Smouse 2006). ANOSIM produces a measure called R global, which varies between −1 and 1, with zero meaning no separation between groups and 1 and −1 meaning complete separation. The null hypothesis of no differences between members of the two species was tested by randomly placing individuals in groups as part of a Monte Carlo permutation procedure (Clarke and Gorley 2001).

### Hybrid identification analyses

To explore the hypothesis of hybridization between *D. andersoni* and *D. variabilis*, we used the program Structure v.2.3.3 (Pritchard et al. 2000) to perform an assignment test (Pearse and Crandall 2004). We used the AFLP presence/absence data, which represent variation in nuclear DNA. All samples were included in the analysis. The *a priori* number of populations (*K*) was set to two, corresponding to each parental species. An admixture model was selected using a burn-in of 25,000 permutations followed by 100,000 repetitions. All runs demonstrated convergence before the end of the burn-in, suggesting good performance of the Markov chain Monte Carlo method. Based on the population analyses of AFLPs, ticks from pure populations were expected to have extremely high assignment probabilities (Q-values >95%). In contrast, hybrid individuals were expected to display much lower Q-values. To be conservative, hybrids were classified as those individuals with assignment probabilities between 50% and 90%. We then tested a wider range of *K* values (1–10) to determine whether putative hybrids clustered separately from either parent species. This wider analysis was also useful for the study of population structure (below), which we confirmed with the Δ*K* method (Evanno et al. 2005).

The NNewHybrids software v1.1 Beta (Anderson and Thompson 2002; Anderson 2008) was used as a second method to confirm the presence of hybrids in our dataset. This software assigns individuals based on the proportion of alleles from the two parental species. In this study, genotype frequency classes were defined only for the first two hybrid generations. As such, individuals were assigned to F_1_, F_2_, or as backcross to either parental species. According to Anderson (2008), the software performs better when pure representatives of the parent species are specified a priori. To ensure proper assignment of individuals, a subset of *D. andersoni* was chosen from locations in our dataset that had the highest likelihood of being “pure” populations (*n* = 32, from Placidia Butte, OR, where no *D. variabilis* occurred). We did the same for *D. variabilis* (*n* = 30, from Pompey's Pillar, MT, where no *D. andersoni* were found). Given the large number of loci (148), a burn-in period of 75,000 repetitions was defined, and 100,000 iterations were run thereafter.

In the AFLP analyses, we identified thirteen loci specific to *D. andersoni,* but none specific to *D. variabilis*. We examined the distribution of these loci among the putative hybrids and pure *D. andersoni* and *D. variabilis* individuals. We compared the occurrence means using an ANOVA and an a posteriori Tukey test. We expected a lower occurrence of these alleles in the putative hybrids than in pure *D. andersoni* if genetic material is introgressing from *D. variabilis* individuals. All 13 alleles were absent in all *D. variabilis* samples.

We performed an admixture analysis to test if genotype frequencies within admixed populations departed from neutral expectations. For this purpose, we used the genomic clines method as described in Gompert and Buerkle (2009) and implemented in Introgress, an R-based script (Gompert and Buerkle 2010). This analysis assumes the existence of an admixture population with two parent “pure” populations and generates genomic clines (regression of observed and expected genotypes in one locus across a genome-wide admixture gradient) (Gompert and Buerkle 2010; Luttikhuizen et al. 2012).

For all the putative hybrid individuals, a portion of the mitochondrial 16S gene was sequenced to determine the female parent species and whether directionality was important in hybrid crosses. We used published primers (Norris et al. 1999) to amplify a 454 base fragment of the mitochondrial 16s. The fragments were TA cloned (TOPO® TA Cloning® Kit for Sequencing, Life Technologies, Carlsbad, CA) and 3–6 clones from each tick were sequenced (BigDye® Terminator Cycle Sequencing Kit, Life Technologies; Applied Biosystems 3130xl Genetic Analyzer). Sequences from multiple clones were assembled into a consensus sequence for each tick using SeqMan Pro (Lasergene, DNA Star Inc., Madison, WI). The consensus sequences from each tick were used in a BLAST search of the GenBank database and all were a perfect match for sequences identified in GenBank as *D. andersoni*.

### Population genetics analyses

The number of polymorphic loci, expected heterozygosity, and overall mean expected heterozygosity were estimated for each population using the package Genalex v.6.3 (Peakall and Smouse 2006). Also, given the dominant nature of AFLPs, Hardy–Weinberg frequencies were assumed for all loci. To evaluate the levels of genetic population differentiation (Φ_ST_) in each species, we performed an Analysis of Molecular Variance (AMOVA) as defined by Excoffier et al. (1992) using Arlequin 3.5.1.2 (Excoffier and Lischer 2010). This software package was also used to calculate pairwise Φ_ST_ population values. To test for isolation by distance (IBD) between populations, we used the RELATE function in Primer v.5.2.9 (Clarke and Gorley 2001). This procedure tests for correlations between two matrices using the Spearman rank measure *ρ*, and is equivalent to a rank Mantel test. In this case, a genetic distance matrix and a linear geographic distance matrix were compared. To test the null hypothesis of no relationship between matrices (*ρ* = 0), 9999 permutations were performed.

## Results

### AFLP analysis

A total of 148 AFLP loci were scored unambiguously for 235 individuals from nine populations of *D. andersoni* and 64 individuals from three populations of *D. variabilis* (Table 1). All of these individuals had unique AFLP profiles, and the overall genotyping error rate was 2.53%. Eleven loci (seven in *D. andersoni* and four in *D. variabilis*) showed weak evidence of linkage disequilibrium. However, none of the 11 loci exhibited significant LD in more than half of the test populations. We ran all subsequent analyses in this study with and without the 11 loci and found minimal changes in the final results. Thus, LD does not appear to be a problem and we present results generated with all 148 loci.

An analysis of similarity (ANOSIM) demonstrated that these species are clearly separated with AFLP markers (*R* = 0.966, *P* = 0.001). Population polymorphism levels varied between 50% and 95.95% for *D. andersoni* and between 65.54 and 78.38% for *D. variabilis* (Table 2). Values of mean heterozygosity estimates were *H*_E_ = 0.284 (SE = 0.050) for *D. andersoni* and *H*_E_ = 0.232 (SE = 0.009) for *D. variabilis* (Table 2).

**Table 2:** Summary of hybrids and genetic diversity in sampled populations of *Dermacentor andersoni* and *D. variabilis*. The count and percentage of hybrids are provided from the NewHybrids analysis

Species	Population	Sample size	Number of hybrids (%)	% of polymorphic loci	*H*_E_ (± SE)
*D. andersoni*	Placidia Butte, OR	32	0 (0%)	64.86	0.244 (± 0.017)
	Haeder Rd., WA	8	0 (0%)	50.0	0.190 (± 0.017)
	Shumway Lake, BC	15	0 (0%)	71.62	0.271 (± 0.017)
	Walker Lake, BC	40	3 (8%)	83.78	0.310 (± 0.015)
	Onefour, AB	11	2 (18%)	72.97	0.270 (± 0.016)
	Miles City, MT	56	8 (14%)	93.24	0.326 (± 0.013)
	Lake Como, MT	53	10 (19%)	95.95	0.328 (± 0.013)
	Corwin Springs, MT	7	2 (29%)	72.97	0.294 (± 0.017)
	Lolo Pass, ID	13	6 (46%)	81.76	0.323 (± 0.015)
*D. variabilis*	Sprague, WA	19	0 (0%)	65.54	0.212 (± 0.017)
	Miles City, MT	5	0 (0%)	78.38	0.276 (± 0.015)
	Pompey's Pillar, MT	40	0 (0%)	75.68	0.209 (± 0.015)

### Hybrid identification

**Figure 2:**
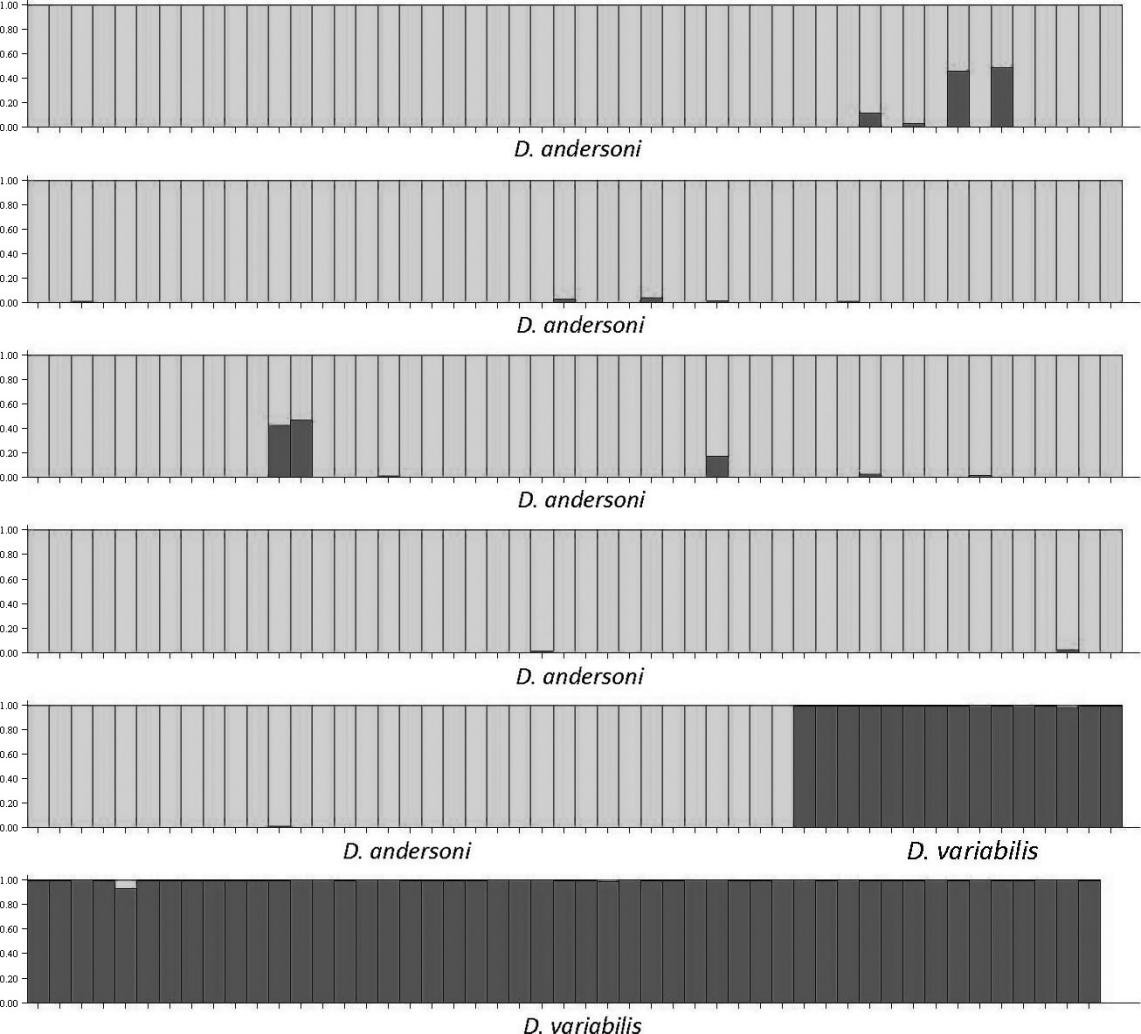
Structure assignment test results assuming the number of groups is *K* = 2. Light gray represents *Dermacentor andersoni* and dark gray, *D. variabilis*. Putative hybrids are represented as a mixture of both species.

Results of the assignment test in Structure (Pritchard et al. 2000) found 18 ticks that are a genetic mixture of the two parent species, with only moderate support (Q = 50–90%) for assignment to *D. andersoni* (Fig. 2). These putative hybrids were found in six of the nine sampled populations: Lolo Pass, ID; Corwin Springs, MT; Lake Como, MT; Miles City, MT; Onefour, AB; and Walker Lake, BC. In tests where *K* > 2, the putative hybrids always demonstrated admixture and in no instance clustered as a separate genetic group. We also ran Structure after removing the 13 loci specific to *D. andersoni* (below) and recovered very similar admixture patterns for the same 18 ticks. The NewHybrids analysis identified these same 18 individuals plus additional 13 potential hybrids that were not identified by Structure. These 13 individuals were collected from the same six populations mentioned above except for Corwin Springs, MT. The NewHybrids analysis estimated zero F_1_ individuals, four F_2_ individuals, 27 backcrosses to *D. andersoni,* and zero backcrosses to *D. variabilis*. In no instances did Structure or NewHybrids predict genetic admixture back into the *D. variabilis* populations.

Significant differences in mean count for thirteen species-specific loci to *D. andersoni* were found among *D. andersoni, D. variabilis,* and the 31 putative hybrid samples (*F* = 457.51, *P* < 0.0001). In all 13 cases, these alleles are specific to *D. andersoni*, not found in *D. variabilis*, and found sporadically in the putative hybrids. A posteriori comparison of all pairs of means showed differences among the three groups of samples (*α* = 0.05). Consistent with the above results, all putative hybrids had 16s mtDNA sequences characteristic of *D. andersoni* (data not shown).

Hybridization should result in loci that deviate from neutral expectations and either facilitate (positive selection) or interfere (negative selection) with genomic introgression between species. In our Introgress analysis, 23 loci (15.5%) showed positive selection, whereas 12 loci (8.1%) demonstrated negative selection as defined by Luttikhuizen et al. (2012). This may suggest that some loci are favored during hybridization between *D. andersoni* and *D. variabilis*, whereas others are selected against.

### Population genetic structure

Genetic differentiation among populations was significant for *D. andersoni* (Φ_ST_ = 0.110, *P* = 0.001). This result suggests moderate genetic connectivity among populations of this species (Table 3). A similar pattern was found in the Δ*K* analysis of Structure, which estimated six genetic groups of *D. andersoni*. A comparison between geographic and genetic distance found that IBD is low within these *D. andersoni* populations (rank Spearman correlation *ρ* = 0.066, *P* = 0.001). In light of the low IBD value, we tested the removal of potential hybrids from the AMOVA to determine if the presence of hybrids was determining the observed population structure. This procedure produced almost no change in Φ_ST_ (0.108, *P* = 0.001).

**Table 3:** Analysis of molecular variance (AMOVA) table and Φ_ST_ values for *Dermacentor andersoni* and *D. variabilis*

	df	Var	%Var
*D. andersoni* Φ_ST_ = 0.110 (*P* = 0.001)			
Among populations	8	2.408	11
Within populations	226	19.415	89
Total	234	21.823	100
*D. variabilis* Φ_ST_ = 0.304 (*P* = 0.001)			
Among populations	2	6.501	30
Within populations	62	14.866	70
Total	64	21.367	100

Significant population structure was also found for *D. variabilis* (Φ_ST_ = 0.304, *P* = 0.001, Table 3). This high level of genetic differentiation might be due to the effect of large geographic distances among populations or strong founder effects upon establishment. A strong IBD was found among these western populations of *D. variabilis* (rank Spearman correlation *ρ* = 0.729, *P* = 0.001). However, this estimate is based on just three populations at the extreme edge of the distribution of this species.

## Discussion

### Hybrids

We report for the first time the natural occurrence of cryptic hybrids between *D. andersoni* and *D. variabilis*. Hybrids between these two species have been described previously in laboratory experiments (Oliver et al. 1972), but not in the wild. A close look at all of our samples revealed 31 putative hybrid and backcross individuals in six populations that originally were considered to be purely *D. andersoni* (Lolo Pass, Corwin Springs, Lake Como, Miles City, Onefour, and Walker Lake). These samples clearly show levels of admixture in their AFLP profiles, which are most likely the result of hybridization between the two tick species. Four individuals (two from Lolo Pass and two from Corwin Springs) were identified as F_2_ generation hybrids. The remaining 27 individuals of the possible hybrids presented different degrees of backcrossing to *D. andersoni*. We discovered 13 loci specific to *D. andersoni*. As expected, these markers display a lower count in the putative hybrids than in pure *D. andersoni* individuals. This suggests that some of these alleles are being lost due to hybridization between these two species. However, a third species of *Dermacentor* tick, *D. albipictus,* is also found in the sampled regions (Bishopp and Trembley 1945) and thus may also be a candidate for hybridization with *D. andersoni*. However, because *D. albipictus* is a one-host tick whose seasonality is distinctly different from that of either *D. variabilis* or *D. andersoni,* it is not a likely candidate for natural hybridization with either of these species. The most likely explanation of our data is that hybridization is happening between *D. andersoni* and *D. variabilis*. We hypothesize that, given the recent range expansion of *D. variabilis* toward the west (Stout et al. 1971), these new populations are being partially absorbed by hybridization to *D. andersoni*. We suspect that hybridization events are more likely to occur in areas where the numbers of available *D. variabilis* mates are low. Backcrosses to *D. andersoni* are more likely to occur because the more rare *D. variabilis* or hybrids are present in a background of abundant *D. andersoni*.

Hybridization of ticks is possible when species overlap in their natural ranges, habitat, and/or host use. Importantly, adults must be in a reproductive state during the same time of year to facilitate interspecific crosses. In the genus *Dermacentor*, hybridization studies have been performed for two sets of species that share these characteristics: *D. marginatus* and *D. reticulatus* (Zahler and Gothe 1997), and *D. variabilis* and *D. andersoni* (Oliver et al. 1972; Dergousoff and Chilton 2007). *D. reticulatus* and *D. marginatus* share partial range overlap, host usage, and similar morphology (Zahler et al. 1995). In experimental reciprocal crosses between these two species, all females engorged and laid eggs (Zahler and Gothe 1997). However, females resulting from interspecific matings were smaller and laid fewer eggs, which, in the end, were nonviable. Reproductive isolation between the two species was confirmed with the use of ITS2 sequencing, which showed that *D. reticulatus* and *D. marginatus* had species-specific genotypes (Zahler et al. 1995).

*D. andersoni* and *D. variabilis* are found sympatrically in several areas in central North America, which creates the potential for hybridization between the species (Dergousoff and Chilton 2007). However, no evidence for hybridization was found in two sympatric populations of *D. andersoni* and *D. variabilis* in Saskatchewan using an ITS2 marker (Dergousoff and Chilton 2007). On the other hand, laboratory experiments have clearly demonstrated the viability of hybrids between *D. variabilis* and *D. andersoni* (Oliver et al. 1972). In this study, only crosses between *D. andersoni* males and *D. variabilis* females produced viable eggs, which is in contrast to our observations. All of the putative samples in our study have a mtDNA signature characteristic of *D. andersoni*, suggesting that viable crosses in the wild are happening between *D. andersoni* females and *D. variabilis* males, but not the other way around. In the laboratory, crosses between F_1_ males and females and backcrosses between hybrid males with either parent species were unsuccessful (Oliver et al. 1972). In spite of this, our data support the viability of backcrosses to *D. andersoni*. In the Oliver et al. (1972) experiments, no crosses between hybrid F_1_ females and either parent species were performed. However, our data suggest that it is via this route that hybrids are likely to be maintained in the wild.

More in-depth investigations are required to explore the potential for selection (Anderson and Stebbins 1954) that arises from the introgression of new genetic material into the *D. andersoni* genomic pool. The Introgress analysis suggests that selection may be acting on certain parts of the genome. This study reveals genetic structure among populations with and without hybridization, which could get stronger if introgression from *D. variabilis* continues to happen. Also, characteristics of hybrid vectors, such as host preference, habitat use, and ability to transmit specific pathogens, remain unknown. In *Culex* mosquitoes, it has been observed that hybrids of two *Culex pipiens* biotypes (*Culex pipiens f. molestus* and *f. pipiens*) broaden their host preferences in a hybrid zone, feeding on both mammals and birds (Byrne and Nichols 1999; Kilpatrick et al. 2007). An important consequence is the potential for these hybrid mosquito vectors to play a larger role in the transmission of West Nile virus to humans. Another case of gene introgression is found in *Anopheles* mosquitoes where evidence has been found of an gene exchange between *A. gambiae* and *A. arabiensis* (Besansky et al. 2003). It is considered that *A. gambiae* acquired the ability to expand its range into arid environments due to the exchange of genetic material with *A. arabiensis*.

In light of these examples, it seems possible that hybridization between *D. andersoni* and *D. variabilis* could broaden the range of environmental conditions in which hybrids can survive. For example, the preferences of *D. andersoni* for semiarid grasslands could be expanded to include deciduous forest and allow it to start moving east into the range of *D. variabilis*. Another possible outcome is the extension of questing period of *D. andersoni*. Seasonal activity of ticks has proven to be a relevant factor in the transmission of pathological agents. For example, it is the 2-year phenology of *Ixodes scapulari*s, with nymphs from the previous generation occurring in the spring before the larvae of the next generation that is responsible for the success of this species as a vector of Lyme disease spirochetes (*Borrelia* spp.) (Spielman et al. 1985; Wilson and Spielman 1985). In the northwestern region where these ticks were collected, *D. andersoni* adults typically quest from early spring into late May, whereas *D. variabilis* adults are seeking hosts from late spring into late June (Scoles unpubl. data). The potential for hybrids to survive and quest across a longer transmission season could have important implications for pathogen transmission. Hybridization could also be a mechanism for moving nontransmissible symbionts between these two species and may explain why the non-transmissible *Rickettsia peacockii*, a symbiont of *D. andersoni*, has been found in both species (Scoles unpubl. data). This may also have implications for vector competence as it has been suggested that the microbiome of a tick can affect its vectoral capacity. These and other potential effects on their role as vectors provide a logical focal point for investigating the range expansion, hydridization, and subsequent introgression of *D. variabilis* genes into populations of *D. andersoni*.

### Population structure

Long-distance dispersal in most ectoparasites depends entirely on the movement of their hosts. For this reason, gene flow and population structure levels are largely dependent on the type of host(s), the level of host specificity, and the ecology of each species involved (Kain et al. 1999). *D. andersoni* is a three-host tick that must quest for a new host at each life stage. This species exhibits little host specificity and parasitizes a broad range of terrestrial mammals (Burg 2001; James et al. 2006). Given the relatively limited dispersal of terrestrial hosts (compared with highly vagile species like birds) and our widespread samples, we expected to find genetic divergence across the northwest intermountain region. Our results from *D. andersoni* generally support these predictions, with moderate population structure and low isolation by distance.

Genetic differentiation in other three host ticks sampled across their geographic range demonstrate *F*_ST_ values between 0.040 and 0.329 (Hilburn and Sattler 1986; Kain et al. 1997, 1999; Lampo et al. 1998; Qiu et al. 2002). This wide range suggests that genetic divergence may be difficult to predict based on life cycle alone. Also, comparisons of divergence values are not always straightforward because of the differences in molecular markers and analytical methods used to evaluate differentiation. The most comparable work to the *D. andersoni* case is that of Kain et al. (1997, 1999) who sampled *Ixodes pacificus* across western North America, including a disjunct population in Utah. Using allozymes, moderate population structure with no isolation by distance was found, although most of the structure was determined by one locus (*F*_ST_ = 0.142) (Kain et al. 1997). Further exploration using mtDNA sequences of the cytochrome oxidase III gene (COIII) within a smaller subset of samples revealed the genetic isolation of the disjunct population in Utah, yet in the absence of isolation by distance (Kain et al. 1999). Levels of population structure in *D. andersoni* (Φ_ST_ = 0.110) and the significant but low value of isolation by distance (*ρ* = 0.066) are comparable to those found in *I. pacificus* (Kain et al. 1997, 1999).

Past work on two populations of *D. andersoni* found on different habitats (montane and prairie) demonstrated the potential for high levels of population differentiation within this species (Lysyk and Scoles 2008). Despite an *F*_ST_ estimate of 0.49 using single nucleotide polymorphisms in a 1.6 kb mtDNA fragment that encompassed the 16S and 12S genes, reciprocal cross experiments found only limited reproductive barriers. Comparatively, our pairwise Φ_ST_ values were 3–4 times lower among the three Canadian populations (Table 4), which suggests that gene flow is higher in *D. andersoni* than initially reported by Lysyk and Scoles (2008). These three locations covered a similar geographic spread to the original collections of Lysyk and Scoles. It is possible that other factors besides geographic distance play a role in determining the genetic population structure observed in this dataset, and unknown differences between prairie and montane populations, including differences in the host assemblages they parasitize, could be an important driver of this pattern.

**Table 4:** Pairwise Φ_ST_ values between *Dermacentor andersoni* populations

Population	Corwin Springs, MT	Haeder Rd., WA	Lake Como, MT	Lolo Pass, ID	Miles City, MT	Onefour, AB	Placidia Butte, OR	Shumway Lake, BC
Haeder Rd., WA	**0.12363**							
Lake Como, MT	**0.07490**	**0.11054**						
Lolo Pass, ID	**0.01932**	**0.14111**	**0.10689**					
Miles City, MT	0.01932	**0.12896**	**0.04318**	**0.09614**				
Onefour, AB	0.04657	**0.17975**	**0.08832**	0.06664	0.02364			
Placidia Butte, OR	0.15565	0.05053	**0.19236**	**0.22350**	**0.18166**	**0.24474**		
Shumway Lake, BC	**0.06064**	**0.07906**	**0.04795**	**0.11789**	**0.07009**	**0.13193**	**0.13046**	
Walker Lake, BC	**0.12206**	0.02355	**0.11171**	**0.15819**	**0.12013**	**0.16202**	**0.09607**	**0.11998**

Statistically significant values after a Bonferroni correction values are presented in bold type.

The level of genetic differentiation in *D. variabilis* suggests that little gene flow has occurred among these populations. Even the two more closely situated populations in Montana (Miles City and Pompey's Pillar) were genetically different (Table 5), indicating a lack of genetic admixture. Polymorphism levels were also low for the three *D. variabilis* populations (Table 2). This is consistent with sampling at the edge of a species' range, where disjunct populations are expected to present lower marker polymorphism and greater genetic differentiation. This westward movement of *D. variabilis* was first reported in the early 1970s, when it was first described in Washington and Idaho (Stout et al. 1971). This migration is thought to be mediated by the movement of humans traveling with their pets, especially dogs, along interstate highways (Scoles pers. obs.). Therefore, two other factors, in addition to isolation, may contribute to the rapid increase in population structure. First, *D. variabilis* transplants may originate from a wide variety of source material in central and eastern North America. Unintentional translocations might simply reflect the genetic variation found in widely separated source populations. Second, female ticks can lay more than 6000 eggs per reproductive season, and only a few individuals are needed to establish a new population. Under these conditions, strong founder effects are to be expected and could explain the low levels of gene flow observed in our results. Further examination of these recent populations is needed to understand whether *D. variabilis* is ecologically established in this region and not simply repopulated each year by humans.

**Table 5:** Pairwise Φ_ST_ values between *Dermacentor variabilis* populations

Population	Sprague, WA	Miles City, MT
Miles City, MT	**0.25200**	
Pompey's Pillar, MT	**0.36747**	**0.13904**

Statistically significant values after a Bonferroni correction values are presented in bold type.
